# Progressive Pulmonary Cystic Disease in Autoimmune Hepatitis and Rheumatoid Arthritis: A Rare Association

**DOI:** 10.7759/cureus.108200

**Published:** 2026-05-03

**Authors:** Havish S Kantheti, Kevin T Huynh, Nina Kanase, Morad H Amar, Lavanya Srinivasan

**Affiliations:** 1 Internal Medicine, Baylor Scott & White Health, Fort Worth, USA; 2 Internal Medicine, Baylor Scott & White Medical Center, Fort Worth, USA; 3 Internal Medicine, Baylor Scott & White All Saints Medical Center, Fort Worth, USA; 4 Pulmonology, Baylor Scott & White All Saints Medical Center, Fort Worth, USA

**Keywords:** autoimmune hepatitis, pulmonary arteriovenous malformation, pulmonary cystic disease, rheumatoid arthritis, tumor necrosis factor inhibitors

## Abstract

Pulmonary cystic lung disease is an uncommon manifestation of systemic autoimmune disorders. Rheumatoid arthritis (RA) is most often associated with interstitial lung disease, whereas pulmonary involvement in autoimmune hepatitis (AIH) is rare. We describe an 82-year-old woman with longstanding RA and AIH who demonstrated progressive bilateral pulmonary cystic disease over a 15-year period, complicated by acute hypoxemic respiratory failure and the new identification of a pulmonary arteriovenous malformation. Interval imaging revealed progression of thin-walled pulmonary cysts and fibrotic changes compared with prior studies. This case highlights a rare overlap of autoimmune conditions associated with cystic lung disease and emphasizes the importance of longitudinal imaging, medication review, and multidisciplinary management in patients with complex autoimmune disease presenting with dyspnea.

## Introduction

Pulmonary involvement is a well-recognized extra-articular manifestation of rheumatoid arthritis (RA), affecting up to 60%-80% of patients when subclinical disease detected on high-resolution computed tomography (CT) is included [1]. The most common pulmonary manifestations of RA include interstitial lung disease (particularly usual interstitial pneumonia and nonspecific interstitial pneumonia patterns), airway disease, pleural effusions, and rheumatoid nodules. Diffuse cystic lung disease (DCLD) is defined by the presence of multiple thin-walled, air-filled parenchymal lucencies, distinct from cavities, emphysematous blebs, and honeycombing [2]. Progressive cyst formation may lead to recurrent pneumothorax, declining lung function, and respiratory failure. Cystic lung disease is distinctly uncommon in RA and is rarely described outside of isolated case reports and small series, making its natural history, pathogenesis, and optimal management poorly understood. Importantly, pulmonary manifestations may precede the formal diagnosis of RA by years, and subclinical autoimmune processes may be operative long before articular symptoms become clinically apparent [3].

The differential diagnosis of DCLD is broad and includes lymphangioleiomyomatosis, pulmonary Langerhans cell histiocytosis, Birt-Hogg-Dubé syndrome, lymphocytic interstitial pneumonia, follicular bronchiolitis, smoking-related cystic changes, and cystic lung disease associated with systemic autoimmune conditions [2]. Among autoimmune disorders, pulmonary cystic disease is most frequently associated with Sjögren’s syndrome, in which lymphocytic infiltration and airway-centered inflammation may lead to cyst formation through a check-valve mechanism of bronchiolar obstruction [4]. Distinguishing among these entities requires careful integration of clinical context, serologic data, and longitudinal imaging.

Autoimmune hepatitis (AIH) is a chronic immune-mediated liver disease characterized by interface hepatitis, hypergammaglobulinemia, and circulating autoantibodies. AIH may be idiopathic or drug-induced; statin-induced AIH is a recognized entity in which atorvastatin and other hydroxymethylglutaryl-coenzyme A reductase inhibitors trigger an immune-mediated hepatic injury that may persist even after drug discontinuation [5]. While extrahepatic manifestations of AIH are well described, including thyroiditis, inflammatory bowel disease, and various dermatologic conditions, pulmonary involvement is uncommon and not well characterized. When present, pulmonary disease in AIH typically manifests as fibrosing alveolitis, organizing pneumonia, or granulomatous inflammation rather than cystic changes [5]. Chronic autoimmune liver disease may also affect the pulmonary vasculature; hepatopulmonary syndrome, characterized by intrapulmonary vascular dilatations in the setting of hepatic dysfunction, has been described even in the absence of overt cirrhosis [6]. The coexistence of progressive pulmonary cystic disease in a patient with both RA and AIH raises important questions regarding shared immune-mediated pathophysiology.

An additional consideration in patients with autoimmune disease is medication-related pulmonary toxicity. Tumor necrosis factor (TNF)-α inhibitors, which are widely used in RA, have been associated with a broad spectrum of pulmonary complications, including sarcoid-like granulomatous reactions, interstitial lung disease, pulmonary fibrosis, and vasculitis [7-9]. The possibility that biologic therapy may contribute to or accelerate atypical pulmonary manifestations warrants careful evaluation, particularly in patients with preexisting lung pathology. However, a direct causal relationship between TNF-α inhibitors and cystic lung disease specifically has not been established, and the role of medication exposure in cyst progression remains uncertain.

We present the case of an 82-year-old woman with seronegative RA and statin-induced AIH who developed progressive bilateral pulmonary cystic disease over a 15-year period, complicated by acute hypoxemic respiratory failure and a newly identified pulmonary arteriovenous malformation (AVM). To our knowledge, this is the first report describing the triad of progressive pulmonary cystic disease, concurrent pulmonary AVM, and overlapping seronegative RA with drug-induced AIH. This co-occurrence raises important questions about shared immune-mediated pulmonary parenchymal and vascular pathology and underscores the importance of longitudinal imaging and multidisciplinary evaluation.

## Case presentation

An 82-year-old woman with a history of seronegative RA, diagnosed in May 2024 based on clinical findings and magnetic resonance imaging, AIH attributed to atorvastatin use, hypertension, and supraventricular tachycardia managed with sotalol 80 mg twice daily, presented with four days of worsening dyspnea and nonproductive cough. She denied fever, hemoptysis, or pleuritic chest pain. She also reported progressive lower extremity edema and chills, without recent travel or known sick contacts. Her RA had initially been treated with hydroxychloroquine from the time of diagnosis until September 2025, after which she was transitioned to golimumab (Simponi Aria), a TNF-α inhibitor administered as intravenous infusions every two months. She had received her first infusion approximately three months prior to presentation. Of note, her outpatient pulmonologist had previously raised concern in August 2025 regarding the initiation of biologic therapy in the setting of her cardiac history, though her rheumatologist proceeded with treatment. She had a 40-pack-year smoking history, having quit 23 years earlier.

On presentation, she was afebrile and required 5 L/minute of supplemental oxygen. Vital signs indicated a heart rate of 57 beats per minute (on sotalol), along with tachypnea, and an oxygen saturation of 93% while on supplemental oxygen. Physical examination demonstrated diffuse wheezing, scattered inspiratory crackles, and chronic symmetric inflammatory joint changes. No digital clubbing or cyanosis was observed. Jugular venous pressure was not elevated. The abdomen was soft and nontender without hepatomegaly or splenomegaly. No mucocutaneous telangiectasias were identified. Selected laboratory findings are summarized in Table 1. Notable results included leukocytosis, elevated procalcitonin, mild hypoalbuminemia, and a minimally elevated troponin with preserved renal function. Rheumatoid factor and anti-cyclic citrullinated peptide antibodies were negative, consistent with her known seronegative RA. An autoimmune serologic panel obtained in April 2024 as part of the initial diagnostic workup demonstrated a positive anti-Sjögren's-syndrome-related antigen A (SSA)/Ro antibody (quantitative 1.1 antibody index (AI); reference: negative less than 1.0 AI) with an otherwise unremarkable extractable nuclear antigen profile: anti-double-stranded DNA antibody was 1 IU/mL (negative; reference: less than or equal to 4 IU/mL), and anti-centromere, anti-chromatin, anti-Jo-1, anti-ribosomal P, anti-ribonucleoprotein (RNP), anti-Scl-70, anti-Smith, and anti-Sm/RNP antibodies were all negative. Anti-SSB/La antibody was not separately reported. Transthoracic echocardiography demonstrated normal left ventricular cavity size with preserved systolic function (ejection fraction 65%-70% by visual assessment). The right ventricle was dilated with normal systolic function (tricuspid annular plane systolic excursion 2.5 cm). The right atrium was dilated. The left atrium was mildly dilated (4.1 cm, volume index 36 mL/m^2^). There was mild aortic stenosis (aortic valve area 1.7 cm^2^, mean gradient 18 mmHg) and mild mitral annular calcification. The E/e’ ratio was elevated at 23.1, suggesting elevated left ventricular filling pressures. The inferior vena cava measured 2.0 cm with >50% collapse, yielding an estimated right atrial pressure of 3 mmHg. A trivial pericardial effusion was present without chamber collapse. Right ventricular systolic pressure could not be estimated due to an insufficient tricuspid regurgitation jet envelope.

**Table 1:** Selected laboratory findings on admission All values were obtained on admission. BNP was measured by immunoassay. Procalcitonin was measured by chemiluminescent microparticle immunoassay BNP: brain natriuretic peptide

Laboratory parameter	Value	Reference range
White blood cell count	14.2 × 10³/µL	4.5-11.0 × 10³/µL
Hemoglobin	11.8 g/dL	12.0-16.0 g/dL
Platelet count	224 × 10³/µL	150-400 × 10³/µL
Blood urea nitrogen	18 mg/dL	7-20 mg/dL
Creatinine	0.9 mg/dL	0.6-1.2 mg/dL
Albumin	2.8 g/dL	3.5-5.0 g/dL
Procalcitonin	0.88 ng/mL	<0.10 ng/mL
Troponin I	0.05 ng/mL	<0.04 ng/mL
BNP	312 pg/mL	<100 pg/mL

**Figure 1:**
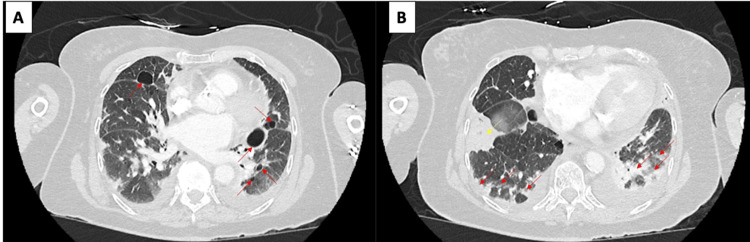
CT of the chest. (A) Bilateral thin-walled pulmonary cysts. (B) Bibasilar fibrotic changes with patchy consolidation and atelectasis Red arrowheads indicate bibasilar fibrotic changes with patchy consolidation and atelectasis; the yellow arrowhead identifies the medial subsegmental right middle lobe pulmonary arteriovenous malformation CT: computed tomography

Computed tomography of the chest revealed bilateral thin-walled pulmonary cysts ranging from approximately 5-25 mm in diameter, distributed predominantly in the lower and middle lobes with some paraseptal and perivascular distribution, with interval progression compared with imaging from 2010, demonstrating an increase in both the number and size of cysts. Associated findings included bibasilar reticulation, traction bronchiectasis, and patchy ground-glass opacification consistent with fibrotic changes. A newly identified enhancing vascular structure was present in the medial subsegment of the right middle lobe, measuring approximately 8 mm, with an identifiable feeding artery and draining vein, consistent with a pulmonary AVM; this finding was not present on prior imaging from 2010 (Figure 1). Given concern for acute hypoxemic respiratory failure in the setting of possible infection and chronic lung disease, she was treated with prednisone 20 mg daily for five days, intravenous ceftriaxone for empiric antimicrobial coverage for three days, and bronchodilator therapy. She was ambulatory with supplemental oxygen as tolerated. Outpatient follow-up was planned to include pulmonary function testing, CT venography or pulmonary angiography, and possible cardiac catheterization for further evaluation of the pulmonary AVM and right heart hemodynamics. Baseline pulmonary function testing was not available from prior encounters.

## Discussion

Pulmonary cystic lung disease in the setting of RA is a rare and underrecognized entity. RA can affect any compartment of the respiratory system, and the lung is among the most common sites of extra-articular involvement [3]. Proposed mechanisms of cyst formation in autoimmune conditions include airway-centered lymphocytic infiltration leading to check-valve bronchiolar obstruction with distal air trapping, follicular bronchiolitis with progressive alveolar wall destruction, and proteolytic injury from chronic inflammation [2,10]. Although these mechanisms are best characterized in the context of Sjögren’s syndrome-associated lymphocytic interstitial pneumonia, they may also be operative in RA-associated cystic disease. Notably, in our patient, the cystic lung disease on imaging predated the formal RA diagnosis by 14 years, suggesting that subclinical autoimmune pulmonary involvement may have been present long before articular disease was clinically recognized. This observation is consistent with the known phenomenon of pulmonary manifestations preceding the formal diagnosis of RA [3]. Prior reviews of thoracic manifestations in RA emphasize that atypical radiographic patterns should prompt careful evaluation for alternative or overlapping etiologies, particularly in patients with complex autoimmune disease or medication exposure [1]. In our patient, the bilateral, lower lobe-predominant distribution of thin-walled cysts with associated fibrotic changes and the absence of nodules or cavitation is atypical for smoking-related cystic disease (typically upper lobe predominant with indiscernible cyst walls) and for pulmonary Langerhans cell histiocytosis (typically associated with nodules in active disease). Although her remote 40-pack-year smoking history introduces the possibility of paraseptal emphysema, the thin-walled cyst morphology, interval progression over 15 years, and bilateral distribution are more consistent with an inflammatory or autoimmune etiology. The autoimmune serologic panel demonstrated a weakly positive anti-SSA/Ro antibody (1.1 AI), with all other extractable nuclear antigens negative. While this finding does not establish a diagnosis of Sjögren’s syndrome, it is noted as part of the broader autoimmune profile and may warrant further outpatient evaluation.

Medication-related pulmonary toxicity further complicates the evaluation of cystic lung disease in patients with autoimmune disorders. TNF-α inhibitors have been associated with a broad spectrum of pulmonary complications, including sarcoid-like reactions, interstitial lung disease, pulmonary fibrosis, and vasculitis [7-9]. Although a direct causal relationship between TNF-α inhibitors and pulmonary cystic disease has not been definitively established, paradoxical immune phenomena and lung injury have been well documented within this drug class. In this case, the patient had received only one infusion of golimumab (Simponi Aria) approximately three months prior to presentation, having been transitioned from hydroxychloroquine. The relatively brief exposure to TNF-α inhibitor therapy makes a direct causal contribution to the cystic changes unlikely; however, the temporal relationship between biologic initiation and worsening respiratory symptoms warrants continued vigilance. The patient’s outpatient pulmonologist had separately raised concern about biologic therapy in the setting of her cardiac history, including supraventricular tachycardia managed with sotalol. Advanced age may further increase susceptibility to medication-related pulmonary toxicity.

A systematic approach to the differential diagnosis of DCLD is essential in this case. Lymphangioleiomyomatosis was considered unlikely given the patient’s age (typically affects premenopausal women) and the absence of renal angiomyolipomas or chylous effusions. Birt-Hogg-Dubé syndrome was considered improbable in the absence of renal tumors, characteristic dermatologic findings (fibrofolliculomas), or family history of recurrent pneumothorax. Pulmonary Langerhans cell histiocytosis was less likely given the absence of pulmonary nodules and the patient’s prolonged smoking cessation interval. Lymphocytic interstitial pneumonia and follicular bronchiolitis, which can occur in association with RA, Sjögren’s syndrome, and other connective tissue diseases and may warrant tissue sampling for definitive diagnosis [10], remained in the differential; however, tissue sampling was not performed during this admission and represents a limitation of this report. Smoking-related cystic disease was considered less likely given the thin-walled cyst morphology, lower lobe distribution, 23-year cessation interval, and the progressive nature of the disease over 15 years on serial imaging.

The identification of a pulmonary AVM adds an additional layer of complexity. Pulmonary AVMs are most commonly congenital or associated with hereditary hemorrhagic telangiectasia; however, idiopathic cases and secondary associations with chronic inflammatory conditions have been described [11]. The absence of mucocutaneous telangiectasias and family history of hereditary hemorrhagic telangiectasia in our patient argues against this diagnosis. An important alternative mechanism to consider is hepatopulmonary syndrome, in which chronic hepatic inflammation or portal hypertension leads to intrapulmonary vascular dilatations and arteriovenous communications [6]. Although the patient’s AIH was attributed to atorvastatin (statin-induced AIH), the duration and severity of hepatic inflammation prior to diagnosis remain unclear, and even drug-induced AIH may produce sufficient chronic hepatic immune activation to affect the pulmonary vasculature. The absence of overt cirrhosis does not exclude subclinical hepatopulmonary vascular remodeling. Right ventricular systolic pressure could not be estimated on echocardiography due to an insufficient tricuspid regurgitation jet envelope, and the planned outpatient cardiac catheterization will be important for characterizing pulmonary hemodynamics. The bilateral right ventricular and right atrial dilation observed on echocardiography, though in the setting of normal estimated right atrial pressure, may represent early or subclinical pulmonary vascular disease, warranting further evaluation. Such lesions may contribute to hypoxemia through right-to-left shunting and may exacerbate symptoms in patients with underlying lung disease. In this patient, the AVM may have compounded hypoxemia and obscured the contribution of progressive cystic lung disease to her presentation. It should be noted that the elevated E/e’ ratio of 23.1, indicating elevated left ventricular filling pressures consistent with diastolic dysfunction, may have independently contributed to this patient’s dyspnea and should be considered in the overall clinical picture.

This case underscores the importance of longitudinal imaging in patients with chronic autoimmune disease and respiratory symptoms. Comparison with prior imaging was essential in establishing the chronicity and progression of pulmonary cystic disease and in distinguishing acute inflammatory changes from longstanding pathology. Specifically, the interval increase in cyst number and size between 2010 and the current presentation confirmed that the cystic changes were progressive rather than static, informing both the diagnostic approach and management decisions regarding continuation of biologic therapy. For patients with overlapping autoimmune disorders receiving biologic therapy, a multidisciplinary approach involving pulmonology, rheumatology, and hepatology is critical to ensure accurate diagnosis, thoughtful medication management, and appropriate long-term surveillance.

Limitations

Several limitations should be acknowledged. Formal evaluation for Sjögren’s syndrome and anti-SSB/La testing were not completed. Baseline pulmonary function testing was unavailable, and a tissue biopsy was not performed to confirm the cystic lung disease etiology. Hepatopulmonary syndrome screening with contrast-enhanced echocardiography was not pursued, and right ventricular systolic pressure could not be estimated due to an insufficient tricuspid regurgitation jet envelope; outpatient cardiac catheterization is planned. The extent of chronic hepatic inflammation prior to AIH diagnosis remains unknown, limiting interpretation of any hepatopulmonary contribution to the AVM.

## Conclusions

This case illustrates the rare association of progressive pulmonary cystic disease in a patient with seronegative RA and statin-induced AIH, complicated by the concurrent identification of a pulmonary AVM. While RA is the most likely driver of the cystic parenchymal changes, the potential contribution of chronic autoimmune hepatic inflammation to pulmonary vascular findings warrants further investigation. The observation that cystic lung disease preceded formal RA diagnosis by over a decade underscores the importance of considering subclinical autoimmune processes in patients with unexplained cystic lung changes. It reinforces the importance of longitudinal imaging comparison, careful medication review in patients receiving biologic therapies, and a multidisciplinary approach to diagnosis and management. Clinicians should maintain a high index of suspicion for atypical pulmonary manifestations in patients with complex autoimmune disease, particularly when multiple potential etiologies coexist.
